# The role of hope in bullying and cyberbullying prevention

**DOI:** 10.3389/fsoc.2025.1576372

**Published:** 2025-07-30

**Authors:** Sameer Hinduja, Justin W. Patchin

**Affiliations:** ^1^Florida Atlantic University, Jupiter, FL, United States; ^2^University of Wisconsin–Eau Claire, Eau Claire, WI, United States

**Keywords:** hope, bullying, cyberbullying, positive psychology, school, youth, adolescence

## Abstract

**Introduction:**

Research is clear that the cognitive-motivational internal asset of hope is significantly related to enhanced life satisfaction and psychological wellbeing. It has also shown promise in preventing participation in a variety of negative externalizing behaviors, especially antisociality, maladaptive coping, and various forms of aggression among young people. The current exploration evaluates the relationship between hope and youth participation in bullying and cyberbullying.

**Materials and methods:**

A nationally representative survey was conducted in spring 2019 among 5,569 U.S. students aged 12–17 (mean age 14.4) to examine bullying and cyberbullying offending, with 2,472 respondents completing Snyder's six-item Children's Hope Scale. The study measured participation in eight forms of school-based bullying and twelve forms of cyberbullying in the previous 30 days, and controlled for demographic variables including age, gender, race, and sexual orientation.

**Results:**

The study found that 16.5% of students participated in school bullying behaviors and 10.7% in cyberbullying behaviors in the previous 30 days. Name-calling was the most common form of school bullying (16%), while making others feel left out was the most frequent form of cyberbullying (10.1%). Statistical analyses revealed that male students were more likely to engage in both forms of bullying. Importantly, higher levels of hope were associated with significantly lower participation in both school bullying and cyberbullying behaviors.

**Discussion:**

Findings indicate that hope has an inverse relationship with school bullying and cyberbullying behaviors among US youth, suggesting that fostering hope could help reduce these forms of interpersonal aggression. Specifically, schools should prioritize hope-building through the cultivation of one-on-one relationships, the use of scenario-based learning, and the implementation of supportive-cooperative interventions.

## The role of hope in bullying and cyberbullying prevention

With the evolution of positive psychology over the last few decades, scholars and practitioners have increasingly seen value in a strengths- rather than risks-based approach to understanding the etiology of deviant behaviors (Seligman and Csikszentmihalyi, 2014; Masten, 2014). Specifically, constructs such as life satisfaction, happiness, self-efficacy, and wellbeing have garnered increased empirical interest in contrast to a historical focus on illness, disorder, and negative psychological and emotional factors (You et al., 2008, p. 447; Taylor et al., 2000). The cognitive-motivational internal asset of *hope* is one construct that encourages a child to reflect on their past experiences, think through what led to them, subsequently be informed and inspired as they tackle their future goals and expectations (Valle et al., 2006).

Research is clear that hope is significantly related to enhanced life satisfaction (Munoz et al., 2017; Cotton Bronk et al., 2009) and psychological wellbeing (Marques et al., 2011; Feldman et al., 2009). It also has shown promise in preventing participation in a variety of negative externalizing behaviors (Snyder et al., 2003), especially antisociality, maladaptive coping, and various forms of aggression among youth (Fite et al., 2017; Cedeno et al., 2010). The current study evaluates the relationship between hope and bullying behaviors by youth at school and online. Before further exploring the origin and potential relevance of hope, we begin by providing a backdrop on the mainstay issues of bullying and cyberbullying. While they have remained prominent public health concerns for years (UNESCO, 2019; National Academies of Sciences, 2016), momentum calling for more actions and solutions from stakeholders worldwide is building.

## School bullying and cyberbullying

The Centers for Disease Control and Prevention (CDC) define bullying as “any unwanted aggressive behavior(s) by another youth or group of youths who are not siblings or current dating partners that involves an observed or perceived power imbalance and is repeated multiple times or is highly likely to be repeated” (Gladden et al., 2014, p. 7). Data from the CDC's Youth Risk Behavior Surveillance System (YRBSS) in 2021 revealed that 15% of high schoolers were bullied at school while between 33 and 44% of middle schoolers (across 13 states who participated in data collection) were bullied at school in the last year (Centers for Disease Control Prevention, 2023). While school bullying victimization data is relatively plentiful, *offending* data are not. One study found that 21% of middle and high school youth in the US participated in bullying at school over the last month (Hinduja and Patchin, 2022). Notably, studies have shown a moderate association between bullying offending and other forms of delinquent and violent behavior including dropping out of school, substance use, fighting, shoplifting, psychopathy, self-harm, and suicide (Ttofi et al., 2016; Baldry, 2014; Walters and Espelage, 2019; Wolke and Lereya, 2015). In addition, bullying perpetration is positively linked with compromised mental health and wellbeing in the form of stress (Konishi and Hymel, 2009; Bru et al., 2001), somatic complaints and problems (Vernberg et al., 2011; Modin et al., 2015), depression (Price et al., 2013), anxiety, and irritability (Campbell et al., 2013; Arslan et al., 2012).

Cyberbullying has been defined as “willful and repeated harm inflicted through computers, cell phones, and other electronic devices” (Hinduja and Patchin, 2024, p. 10), and is generally manifested through cruel, embarrassing, or threatening messages, posts, photos, or videos in social media, gaming, and chat environments. In the US, data from the aforementioned YRBSS revealed that 16% of high schoolers were bullied electronically while between 23 and 35% of middle schoolers were bullied electronically in the last year (Centers for Disease Control Prevention, 2023). Here too, national-level cyberbullying offending data in the US is scarce; one reference point comes from Hinduja and Patchin's (2024) analysis of 2,546 youth in 2021, where 4.9% said they had cyberbullied others in the last month. Research shows that those who cyberbully others struggle with anxiety and depression, and experience more stress, negative emotions, psychosomatic difficulties, traumatic outcomes, lower self-esteem, and poorer self-efficacy (Hinduja and Patchin, 2025; Patchin and Hinduja, 2010; Wong et al., 2014; Sourander et al., 2010; Bergmann and Baier, 2018; Campbell et al., 2013). Moreover, those who participate in cyberbullying are also more likely to engage in school-based bullying, smoking and substance abuse, drunkenness, delinquency, and suicidal ideation (Hinduja and Patchin, 2007; Beckman et al., 2012; Chan and Wong, 2019).

## Hope

Within positive psychology, hope has been characterized not strictly as an emotion (Averill, 2012; Farran et al., 1995) but as an active (as opposed to passive) thinking process comprised of two constructs (Snyder, 1998). The first has to do with the self-perception that one can create a route toward a goal (*pathways*), and the second has to do with mustering and possessing the motivation to travel down that route (*agency*) (Snyder, 2005). Both of these are learned through developmental lessons—with pathways taught through experiences where correlation or causation is drawn (e.g., *this action contributes to that outcome*) and agency taught through observations and realizations that one can engender outcomes for themselves (e.g., *I can make this happen*) (Snyder, 2002). If these thinking processes of pathways and agency have been internalized successfully and regularly during early childhood, those youth will have a higher level of hope than their peers. If not, they will have a lower (i.e., deflated) level of hope as compared to others. Both pathways and agency are required to produce high hope; possessing just one is not sufficient (Snyder, 1994, 1995).

Even though these processes are cognitive and motivational in nature, emotions are still involved in that they reflect perceived levels of hope in the current situation (Snyder et al., 1991). When a child is thwarted from accomplishing a goal, they experience negative emotions. When they successfully achieve a goal, positive emotions result (Snyder et al., 1996). Their emotional state, then, hinges on how well they are doing, or have done, when pursuing goals. This leads to them approaching and engaging with all aspects of their life (e.g., social interactions, relationships, stressors, etc.) with the emotional state that aligns with the level of hope they have accumulated over time. In short, if they have been repeatedly thwarted—and if they have a weakened sense of agency—in accomplishing goals, their hope levels will be low and their emotions will be negative. These negative emotions create pressure for corrective action (Agnew, 1992, 2001) and can accordingly correspond to anti-social, aggressive, and delinquent actions (increased psychopathology -> negative internalizing and externalizing behaviors) (Ostrowsky and Messner, 2005; Broidy, 2001; Maschi et al., 2008). Said another way, a deficiency or absence of hope can contribute to maladaptive behaviors that harm oneself (Carvajal et al., 1998) or others (Hagen et al., 2005).

Our investigation of hope can be situated within a broader social-ecological framework that conceptualizes bullying behaviors as arising from multidirectional relationships between individuals and various systems including family, peer groups, schools, communities, and society. For example, the diathesis-stress model (Swearer and Hymel, 2015) suggests that individual vulnerabilities interact with social and environmental stressors to produce different outcomes. Youth may be involved in differential levels of bullying experiences because of an interplay involving family conditions (e.g., positive parenting in the form of warmth, structure, and autonomy support) (Hinduja and Patchin, 2022), school climate (Hinduja and Patchin, 2012; Wang et al., 2013; Cornell et al., 2015), the deterrent influence of educators (Patchin and Hinduja, 2018; Fernández-Rouco et al., 2022), peer group dynamics (Thornberg, 2015; Van Ryzin and Roseth, 2022), and normative beliefs about the acceptability of certain aggressive behavior (Dillon and Lochman, 2022; Gendron et al., 2011; Wu et al., 2024).

To be sure, hope may interact with many of the aforementioned socio-ecological components; it may mitigate the effects of family stress, influence how youth navigate peer relationships, and shape how youth respond to educational opportunities and school-based social and emotional learning efforts. Snyder (2002) has suggested that adaptive behaviors are both the cause and consequence of hope, and other researchers have shown that those individuals with high levels of hope should have an increased ability to manage uncertainty, adversity, trauma, and strife without resorting to unhealthy coping mechanisms (Goodman et al., 2017; Pan et al., 2021; Lucas et al., 2020; Liu et al., 2017). Understanding how hope functions, then, as a protective factor against bullying behaviors can complement these other approaches and provide another key target area for intervention by relevant stakeholders who work with and care for youth.

Specific to the relationship between hope and bullying, the literature base is sparse and largely based on non-generalizable samples (Sparks et al., 2021). The research that has been done has focused on the role of hope as a *mood* (Dilmaç, 2017) or as *psychological capital* (Cassidy et al., 2014) that can serve to mediate the impact of various stressors (Cleveland and Sink, 2017; Yarcheski et al., 2011; Snyder, 2002) and can promote wellbeing (Marques et al., 2015; Pleeging et al., 2021). Toward this end, some inquiries have identified that hope can mitigate the effects of bullying *victimization* on emotional functioning, and thereby reduce a variety of negative internalized outcomes among youth (such as depression, loneliness, worry, fear, and other indicators of emotional dysfunction) (Zhang et al., 2019; Carney et al., 2019; Hanley and Gibb, 2011; Nixon et al., 2023). With regard to *offending* behaviors, another empirical inquiry involving ~1,000 3rd−6th graders from a rural school district in the US identified that school bullying is positively tied to emotional difficulties, and that higher levels of hope are linked to fewer emotional difficulties (Carney et al., 2019). Notably, the researchers speculated that those who bully others do so because their goals (e.g., gaining social dominance or capital, popularity, or control) are perceived to be not otherwise achievable. Being thwarted from attaining these goals reduces their levels of hope and leads to emotional difficulties—which manifests in increased bullying (Carney et al., 2019).

Scholars have called for additional research to uncover how hope and an ability to look forward to a future of goal attainment positively affects the developmental trajectory of youth (Bell and Jenkins, 1993; Bryant and Ellard, 2015). When viewing these phenomena through a rational choice framework, engaging in bullying or cyberbullying requires meaningful consideration of the positives and negatives of such a choice (both short-term and long-term) (Paternoster and Piquero, 1995; Matsueda et al., 2006). Within this cognitive decision-making process, then, youth with higher levels of hope presumably consider how aggressing against others will potentially compromise their expectations of the person they want to be and the future they wish to have (Oyserman and Markus, 1990; Sparks et al., 2021). As such, they will actively align their actions with their aspirational identity and future goals (Carney et al., 2019, p. 382), and refrain from harmful behaviors toward others.

Given this backdrop and the dearth of research on the role of hope in mitigating problematic externalizing behaviors (Stoddard et al., 2011), we hypothesize an inverse relationship between hope and school bullying as well as between hope and cyberbullying, based on the argument that hope serves as a protective factor in the lives of youth. That is, we expect that youth who have high hope for the future will be less inclined to risk putting that positive future at risk by violating social norms (Toby, 1957). Similarly, those youth who lack hope will be less concerned about the short or long-term consequences of their actions and may be more willing to act impulsively in response to some perceived or actual challenge. In short, we predict the more hope students have, the less likely they are to participate in school bullying or cyberbullying.

## Methodology

Data for the present inquiry came from a survey administered in the spring of 2019 to a nationally-representative sample of English-speaking 12–17-year-old middle and high school students residing in the United States (mean age = 14.4). Parents were contacted via email and given the opportunity to have their child participate in a survey that examined perceptions of, and experiences with, bullying, cyberbullying, and related teen behaviors. Nested age, race, gender, and region quotas, based on U.S. Census parameters, were used to ensure a diverse sample of respondents that was representative of students in the United States. Active parental consent and child assent was obtained. Given these measures to obtain a representative sample, the overall response rate was ~15%.

The total sample included 5,569 respondents. Data for the current study were restricted to the 2,472 respondents who were presented with the Children's Hope Scale. The average age of the subsample was 14.4 (see Table 1). Of these, 50.4% were male, 66.7% White/Caucasian, 13.6% Hispanic, 11% African American, 4.2% multiracial, and 5% another race. The project methodology was approved by the Institutional Review Board of the University of Wisconsin-Eau Claire.

**Table 1:** Sample demographic characteristics (*N* = 2,472).

	**Percent**
**Gender**	
Female	49.2
Male	50.4
Other	0.3
**Age (mean** = **14.4)**	
12	15.9
13	19.4
14	16.9
15	16.2
16	15.2
17	16.4
**Race**	
White/Caucasian	66.7
Hispanic or Latin American	13.6
Black/African American	11.0
Asian	3.2
American Indian or Native	0.7
Multiracial	4.2
Other	0.5
**Sexual orientation**	
Heterosexual	93.5
Not heterosexual	6.5

## Measures

### Bullying offending

Respondents were instructed that bullying is “When someone intentionally and repeatedly harasses, mistreats, or makes fun of another person. But we don't call it bullying when the teasing is done in a friendly and playful way.” The dependent measure of *school bullying offending* represented participation in the previous 30 days as an offender of any of eight different forms of bullying. The varieties of these specific bullying behaviors were informed by Olweus (2007) and are listed in Table 2. The response set for these questions was “never,” “once,” “a few times,” and “many times” (and ranged from 0-3). We combined these experiences into a binary variable with students who reported that they participated in one or more of the eight behaviors two or more times coded as 1, while those who had no experience or just a single experience with bullying were coded as 0 (mean = 0.16; standard deviation = 0.37; Cronbach's α = 0.92).

**Table 2:** Bullying behaviors (*N* = 2,472).

	**Mean**	**Std. dev**.	**Percent**
*School Bullying Scale* (α = 0.92)	0.16	0.37	
I called another student mean names, made fun of or teased him or her in a hurtful way			16.0%
I have taken part in bullying another student or students at school			12.3%
I kept another student out of things on purpose, excluded him or her from my group of friends or completely ignored him or her			11.6%
I spread false rumors about another student and tried to make others dislike him or her			7.2%
I bullied another student with mean names, comments, or gestures with a sexual meaning			6.0%
I bullied another student with mean names or comments about his or her race or color			4.9%
I threatened or forced another student to do things he or she didn't want to do			4.3%
I bullied another student with mean names or comments about his or her religion			4.0%
*One or more of the above, two or more times*			*16.5%*
*Cyberbullying Scale* (α = 0.96)	0.11	0.31	
I said something online that made someone feel left out			10.1%
I posted mean or hurtful comments about someone online			9.3%
I spread rumors about someone online, through text messages, or emails			6.9%
I threatened to hurt someone through a cell phone text message			4.5%
I posted a mean or hurtful picture online of someone			4.4%
I threatened to hurt someone while online			4.4%
I posted mean names or comments online about someone's religion			4.1%
I pretended to be someone else online and acted in a way that was mean or hurtful to them			4.0%
I posted mean names or comments online about someone's race or color			3.7%
I posted mean names, comments, or gestures about someone with a sexual meaning			3.5%
I posted a mean or hurtful video online of someone			3.5%
I created a mean or hurtful web page about someone			3.3%
*One or more of the above, two or more times*			*10.7%*

Percent of respondents who participated in the behavior at least once in the previous 30 days.

The second dependent variable, *cyberbullying offending*, represented the respondent's participation in the previous 30 days as a perpetrator of any of twelve different forms of online bullying (listed in Table 2). The response set for these questions was the same as the school bullying questions. Here again, we combined these experiences into a binary variable; students who reported participation in one or more of the twelve behaviors two or more times were coded as 1, while those who had no or only one experience with cyberbullying were coded as 0 (mean = 0.11; standard deviation = 0.31; Cronbach's α = 0.96).

### Hope

We used Snyder et al.'s (1997) six-item Children's Hope Scale, which assesses two key components. The first is *agency*, which reflects a child's belief in their ability to start and continue actions toward achieving goals. The second is *pathways*, which measures their capacity to find different ways to reach those goals. The former is assessed with statements including “I think I am doing pretty well,” “I am doing just as well as other kids my age,” and “I think the things I have done in the past will help me in the future.” The latter is measured with items including “I can think of many ways to get the things in life that are most important to me,” “When I have a problem, I can come up with lots of ways to solve it,” and “Even when others want to quit, I know I can find ways to solve the problem.” The response set was a six-point Likert scale including the following: “none of the time,” “a little of the time,” “some of the time,” “a lot of the time,” “most of the time,” and “all of the time.” Responses were combined into a summary scale that ranged from 0 to 30 with higher numbers indicating more hope (mean = 21.3; standard deviation = 6.2; Cronbach's α = 0.93). According to the use of this scale across multiple samples of hundreds of children, the average level of hope is 25 (Snyder et al., 1997).

### Demographic covariates

As referenced above, age, gender, race, and sexual orientation were included as controls in the models to account for any influence they might have on school bullying and cyberbullying behaviors (see Table 1).

## Data analysis

Statistical analyses were conducted using SPSS (version 29.0). We first computed descriptive statistics to understand the characteristics of the sample and nature and extent of bullying and cyberbullying offending by youth in the sample. Next, we estimated four multivariate logistic regression models exploring the relationship between hope and school bullying and cyberbullying. We first examined the influence of demographic variables (gender, race, age, and sexual orientation) on school bullying offending. We then added hope as an explanatory variable in a separate model. Similarly, we examined the influence of demographic variables on cyberbullying offending. Then, we added hope into the cyberbullying model. Logistic regression as an analytic technique was deemed appropriate for the research questions given the dichotomous dependent variables, and because it is also relatively intuitive to interpret results. In all models, statistical significance was determined using a 95% confidence interval (two-tailed tests) and missing data (fewer than 5 cases per variable) were excluded listwise.

## Results

Table 2 shows that 16.5% of students in our sample reported that they had participated in one or more of the eight school bullying behaviors, two or more times, in the previous 30 days. The most commonly reported behavior was “I had called another student mean names, made fun or teased him or her in a hurtful way.” Sixteen percent of respondents said they had done that at least once. About 4% of respondents reported that they had threatened or forced another student to do things he or she didn't want to do. With respect to cyberbullying, 10.7% of students in our sample reported that they had participated in one or more of the twelve cyberbullying behaviors two or more times. The most frequently reported form of cyberbullying was “I said something online that made someone feel left out” (10.1% said they had done this at least once in the previous 30 days). Posting mean or hurtful comments online about someone else was also a commonly reported form (9.3%). Fewer than 5% of students had threatened to hurt someone while online.

Results of the logistic regression analyses are presented in Table 3. Male students were significantly more likely than females to have participated in school bullying (Model 1: Exp[*B*] = 1.63, *p* < 0.001) and cyberbullying (Model 3: Exp[*B*] = 1.37, *p* < 0.01). White students were significantly less likely to participate in cyberbullying (Model 3: Exp[*B*] = 0.74, *p* < 0.05). There were no statistically significant differences in participation in school bullying or cyberbullying by age or sexual orientation. When hope was included in the models (Model 2 and Model 4), there were no substantive changes in the relationship between the demographic variables and bullying. Hope was significantly and negatively associated with participation in both school bullying (Model 2: Exp[*B*] = 0.95, *p* < 0.001) and cyberbullying (Model 4: Exp[*B*] = 0.96, *p* < 0.001). That is, students who reported higher levels of hope were less likely to have participated in bullying at school or online.

**Table 3:** Logistic regression coefficients representing effects of hope on school bullying and cyberbullying.

	**D.V**. = **School bullying**		**D.V**. = **Cyberbullying**	
	**Model 1**	**Model 2**	**Model 3**	**Model 4**
	**B(SE) Exp(B)**	**B(SE) Exp(B)**	**B(SE) Exp(B)**	**B(SE) Exp(B)**
Male	0.49 (0.11)^***^ 1.63	0.48 (0.11)^***^ 1.62	0.31 (0.13)^**^ 1.37	0.30 (0.13)^*^ 1.35
White	−0.14 (0.11) 0.87	−0.19 (0.11) 0.83	−0.30 (0.13)^*^ 0.74	−0.34 (0.13)^*^ 0.71
Age	−0.04 (0.03) 0.96	−0.04 (0.03) 0.97	0.03 (0.04) 1.03	0.04 (0.04) 1.04
Heterosexual	0.10 (0.23) 1.11	0.29 (0.24) 1.34	−0.26 (0.25) 0.77	−0.13 (0.25) 0.88
Hope		−0.06 (0.01)^***^ 0.95		−0.04 (0.01)^***^ 0.96
Constant	−1.30 (0.52)	−0.36 (0.54)	−2.25 (0.60)^***^	−1.61 (0.63)^*^
Nagelkerke *R*^2^	0.016	0.043	0.007	0.021

^*^p < 0.05; ^**^p < 0.01; ^***^p < 0.001 (two-tailed).

## Discussion

Results of the current research show that, as theorized, hope is inversely related to school bullying and cyberbullying offending among US youth. Youth may act aggressively toward others when they struggle to construct positive ideas about their future, and lack the ability and resolve to achieve the goals they desire (Chen and Vazsonyi, 2013). Said another way, their lack of hope affects their capacity to rely on, and ability to work toward, positive outcomes and circumstances down the road. Once that is compromised, internal and external controls on their behavior are weakened, freeing them to engage in harmful interpersonal behaviors at school or online.

## Limitations

As with any research endeavor, certain caveats are warranted when considering the results and subsequent implications. While the demographic characteristics of the sample approximate those of the US, there may be uncontrolled-for differences between those who ultimately agreed to participate in the study and those who did not. Another limitation is the cross-sectional nature of the data, and the attendant temporal ordering concerns. Participation in school bullying and cyberbullying also may have been underreported because of the tendency of individuals to provide socially desirable answers (Lee, 1993). Also, some have argued that data stemming from individuals' recollection about the past is inherently unreliable because of the tendency for them to misrepresent or distort facts from a previous time period (Horvath, 1982; Morgenstern and Barrett, 1974). This threat was limited in the current study by asking students to report only on relatively recent experiences (those which occurred in the most recent 30 days).

In addition, the model did not account for critical social and environmental stressors such as familial conflict, school climate, peer group norms, or community-level beliefs about aggression that may moderate or mediate this relationship. Adolescents with low levels of hope may be more susceptible to cyberbullying behaviors in environments where peer groups normalize online aggression or familial instability exacerbates emotional dysregulation. Conversely, supportive school climates or prosocial peer networks may serve to buffer the effects of low levels of hope. It also bears mentioning that the operationalization of hope as a standalone psychological construct risks oversimplifying the ecological pathways to cyberbullying. Normative beliefs across the peer group could serve as a mediator where low levels of hope predisposes youth to adopt attitudes that justify harming others online. Relatedly, family-level factors such as lack of parental supervision and involvement might moderate the relationship by intensifying hopelessness and reducing access to coping resources. Future research should attempt to disentangle whether hope directly predicts cyberbullying or functions within a broader network of socio-ecological influences. Finally, a longitudinal study would clarify causality and illuminate whether variations in hope over time correspond with differences in bullying and cyberbullying offending.

## Policy implications

Teaching and cultivating hope is not prioritized in US school systems, likely given the lack of resources and expertise related to these and other related “soft” skills. This is unfortunate because educational policy advocates strongly recommend such programming given that building such social and emotional assets and cultural competencies is extremely fruitful in improving attendance, academic performance, overall wellbeing, and other key outcomes (Dixson, 2019; Durlak et al., 2011). To the point of the current work, research has shown that levels of hope can be increased even in short-term individual sessions (Feldman and Dreher, 2012), and so counselors and educators should be able to prioritize this strengths-based approach in their work with students to help reduce these aggressive behaviors.

Relatedly, scenario-based experiential learning via role-playing or the discussion of real-world situations has been shown to help youth develop an action plan to solve social and relational problems in healthier ways (Carney et al., 2017; Merrill et al., 2017). Also, supportive-cooperative interventions where students who have bullied others are enlisted to be part of the solution (e.g., specifically tasked with the responsibility to look out for and take care of other students and help to change the social dynamics) (Wachs et al., 2019; Salmivalli, 2010) can be very fruitful in both in the short- and long-term. If their involvement stemmed from thwarted goal attainment and a consequent lack of hope, such inclusive empowerment and assigned leadership may provide a positive, prosocial pathway to what they desired all along (Ellis et al., 2016).

Building hope in youth is likely done more successfully through the use of one-to-one relationship building. Hope therapy (Lopez et al., 2004) is one formal practice comprising of specific components shown to augment hope via weekly sessions in schools (Pedrotti et al., 2008; Lopez, 2013). For example, a therapeutic alliance for building hope is developed through *hope bonding*. Assisting a child to make positive decisions, develop clear and achievable goals, and determine the best pathways to their attainment can occur through *hope-enhancing*. Finally, *hope-reminding* facilitates encouragement through repetition, practice, and the regular cognitive employment of hope in one's daily life (Edwards and McClintock, 2013). If it is not possible to accomplish this in a one-to-one capacity, doing so in groups where two adults work with 8–12 students also materially can increase levels of hope and other cognitive-motivational assets (Marques et al., 2011). It is clear that practices which make meaningful headway in building hope are suffused with intentionality, a deep understanding of youth developmental desires, and actionable, relatable methods to reinforce the relevant skills at hand. We strongly encourage these to be prioritized in communities given the salience of hope in decreasing the likelihood of bullying and cyberbullying perpetration.

## Author's note

Sameer Hinduja, Ph.D.—Sameer Hinduja is a Professor of Criminology and Criminal Justice at Florida Atlantic University. He is recognized internationally for his translational work on the use and misuse of emerging technologies among youth. His research seeks to illuminate how best to promote civility, deter harmful behavior, build healthy communities, and proactively reduce victimization online.

Justin W. Patchin, Ph.D.—Justin W. Patchin is a Professor Criminal Justice at the University of Wisconsin-Eau Claire. For two decades he has been exploring the intersection of teens and technology, with particular focus on cyberbullying, social networking, and sexting. He has written nine books and numerous academic and professional articles on adolescent behaviors online.

## Data Availability

The raw data supporting the conclusions of this article will be made available by the authors, without undue reservation.
